# Long noncoding RNA PXN‐AS1‐L promotes the malignancy of nasopharyngeal carcinoma cells via upregulation of SAPCD2

**DOI:** 10.1002/cam4.2227

**Published:** 2019-06-07

**Authors:** Xiaodong Jia, Po Niu, Cuncun Xie, Hongjian Liu

**Affiliations:** ^1^ Department of Otolaryngology Henan Province People's Hospital of Henan University Zhengzhou China; ^2^ Department of Radiotherapy Henan Province People's Hospital of Henan University Zhengzhou China

**Keywords:** long noncoding RNA, malignancy, nasopharyngeal carcinoma, RNA‐RNA interaction, SAPCD2

## Abstract

Accumulating evidences highlight the critical roles of long noncoding RNAs (lncRNAs) in a variety of cancers. LncRNA PXN‐AS1‐L was previously shown to exert oncogenic roles in hepatocellular carcinoma. However, the expression, role, and molecular mechanism of PXN‐AS1‐L in nasopharyngeal carcinoma (NPC) malignancy remain unknown. Here, we determined that PXN‐AS1‐L is upregulated in NPC tissues and cell lines. Increased expression of PXN‐AS1‐L predicts worse prognosis of NPC patients. PXN‐AS1‐L overexpression promotes NPC cell proliferation, migration, and invasion in vitro, and NPC tumor growth in vivo. PXN‐AS1‐L silencing suppresses NPC cell proliferation, migration, and invasion in vitro. Mechanistically, PXN‐AS1‐L directly interacts with SAPCD2 mRNA 3′‐untranslated region, prevents the binding of microRNAs‐AGO silencing complex to SAPCD2 mRNA, and upregulates the mRNA and protein level of SAPCD2. SAPCD2 is also increased in NPC tissues. The expression of SAPCD2 is significantly positively associated with that of PXN‐AS1‐L in NPC tissues. Gain‐of‐function and loss‐of‐function experiments demonstrated that SAPCD2 also promotes NPC cell proliferation, migration, and invasion. Furthermore, depletion of SAPCD2 significantly reverses the roles of PXN‐AS1‐L in promoting NPC cell proliferation, migration, and invasion in vitro, and NPC tumor growth in vivo. In conclusion, lncRNA PXN‐AS1‐L is upregulated in NPC and promoted NPC malignancy by upregulating SAPCD2 via direct RNA‐RNA interaction.

## 1 INTRODUCTION

Nasopharyngeal carcinoma (NPC) is one of the predominant head and neck cancers which derived from nasopharyngeal (NP) epithelium.^1^ Although radiotherapy with or without neoadjuvant chemotherapy has shown satisfactory treatment results for NPC patients at early stages, most NPC patients at late stages are difficult to treat.^2^ Enhancing the understanding of pathogenic mechanisms of NPC is beneficial for the identification of druggable targets for NPC.

Many genomic abnormalities have been found in NPC, including *ARID1A*, *CDKN2A/B*, *TP53*, *RASSF1*, *SYNE1*, *THY1*, *CCND1*, *PIK3CA*, and so on.^3^, ^4^ Furthermore, many epigenetic variations also contribute to the aberrant gene expression programs which promote NPC malignant progression.^3^, ^5^, ^6^ Among the epigenetic abnormalities, DNA methylation, histone modifications, and noncoding RNAs gradually show their importance in modulating gene expression and controlling NPC progression.^7^, ^8^ Long noncoding RNAs (lncRNAs) is a class of noncoding RNAs with limited protein‐coding capacity and longer than 200 nucleotides in length.^9^, ^10^, ^11^ Transcriptomic sequencing has identified significantly much more number of lncRNAs than that of mRNAs in human cells.^12^ Moreover, many lncRNAs are detected to be dysregulated in various pathological statuses, particular in cancers.^13^, ^14^, ^15^, ^16^ Some of these dysregulated lncRNAs are associated with diagnoses and/or prognoses of cancers.^17^, ^18^, ^19^ In addition, a variety of lncRNAs are demonstrated to play oncogenic or tumor suppressive roles via regulating cancer cell viability, cell proliferation, cell cycle, cell apoptosis, cell migration, cell invasion, cell senescence, tumor growth, metastasis, and so on.^20^, ^21^, ^22^, ^23^, ^24^

The expression pattern and roles of several lncRNAs in NPC have been studied.^8^, ^25^, ^26^, ^27^, ^28^, ^29^, ^30^ LncRNA AFAP1‐AS1 facilitates NPC metastasis via modulating miR‐423‐5p/Rho/Rac pathway.^7^ LncRNA PVT1 is increased in NPC, predicts poor prognosis, and induces radioresistance.^31^ LncRNA LOC284454 is upregulated and correlated with poor outcome in NPC.^32^ LOC284454 promotes NPC migration and invasion through regulating the Rho/Rac signaling pathway.^32^ Although the aberrant expression and roles of these lncRNAs have been reported, other lncRNAs may also participate in the tumorigenesis and development of NPC.

LncRNA PXN‐AS1‐L is a special isoform of lncRNA PXN‐AS1, which was recently reported to be increased in hepatocellular carcinoma (HCC) tissues.^33^ PXN‐AS1‐L was also revealed to promote HCC tumorigenesis via upregulating PXN.^33^ However, the expression, role, and action mechanism of PXN‐AS1‐L in NPC are unknown. In this study, we determined the expression pattern of PXN‐AS1‐L in NPC tissues and cell lines, analyzed the correlation between PXN‐AS1‐L expression levels and survival of NPC patients, and explored the roles of PXN‐AS1‐L in NPC cell proliferation, migration, and invasion, and in vivo NPC tumorigenesis. In addition, the molecular mechanisms responsible for the roles of PXN‐AS1‐L in NPC were also investigated.

## 2 METHODS

### 2.1 Human tissue specimens

The Medical Ethics Committee of the People's Hospital of Henan Province (Zhengzhou, China) reviewed and approved the use of clinical tissue specimens. A total of 72 fresh NPC tissues and 22 fresh noncancerous NP tissues were acquired at the time of diagnosis with written informed consent from the People's Hospital of Henan Province (Zhengzhou, China). All these specimens were diagnosed by histopathological examination. The performance of this study was in accordance with Declaration of Helsinki.

### 2.2 Cell culture

Immortalized human normal NP epithelium cell line NP69 and NPC cell lines SUNE1, CNE1, CNE2, HONE1, and HNE1 were acquired from Sun Yat‐sen University Cancer Center (Guangzhou, China). NP69 cells were maintained in Keratinocyte/serum‐free medium (Invitrogen, Grand Island, NY) supplemented with bovine pituitary extract (BD Biosciences, San Diego, CA). NPC cell lines were maintained in RPMI 1640 medium (Invitrogen) supplemented with 10% fetal bovine serum (Gibco, Grand Island, NY). All these cells were cultured in a humidified incubator containing 5% CO_2_ at 37°C.

### 2.3 Plasmids construction, transfection, and stable cell lines construction

PXN‐AS1‐L full‐length sequences were synthesized by GenScript (Nanjing, China) and cloned into the Hind III and BamH I sites of pcDNA3.1 plasmid (Invitrogen), named as pcDNA3.1‐PXN‐AS1‐L. The PXN‐AS1‐L full‐length sequences were also cloned into the Hind III and BamH I sites of pSPT19 plasmid (Roche, Mannheim, Germany), termed as pSPT19‐PXN‐AS1‐L. SAPCD2 coding sequences were PCR amplified using Platinum^®^ *Pfx* DNA Polymerase (Invitrogen) and the primers 5′‐CCCAAGCTTTATTGTCGCCGTGGGCTGAG‐3′ (sense) and 5′‐GGAATTCATCTGGCAAGGGCGGCAGGAA‐3′ (anti‐sense). The PCR products were cloned into the Hind III and EcoR I sites of pcDNA3.1 plasmid (Invitrogen), named as pcDNA3.1‐SAPCD2. The 3′‐untranslated region (3′UTR) of SAPCD2 mRNA was PCR amplified by Platinum^®^ *Pfx* DNA Polymerase (Invitrogen) and the primers 5′‐CGAGCTCACCCACCCTCTCTGGCTGGAGA‐3′ (sense) and 5′‐GCTCTAGATCGGGGGAACAGGCTTTGCCTAT‐3′ (anti‐sense). The PCR products were cloned into the Sac I and Xba I sites of pmirGLO plasmid (Promega, Madison, WI), termed as pmirGLO‐SAPCD2. cDNA oligonucleotides inhibiting PXN‐AS1‐L or SAPCD2 expression were synthesized by GenePharma (Shanghai, China) and inserted into the GenePharma SuperSilencing^™^ shRNA expression plasmid pGPU6/Hygro, named as sh‐PXN‐AS1‐L or sh‐SAPCD2, respectively. Scrambled shRNA was employed as negative control (NC), termed as sh‐NC. The sequences of the shRNAs were as follows: for sh‐PXN‐AS1‐L, 5′‐CACCGGGATATGCCCAGAGGAAATCTTCAAGAGAGATTTCCTCTGGGCATATCCCTTTTTTG‐3′ (sense) and 5′‐GATCCAAAAAAGGGATATGCCCAGAGGAAATCTCTCTTGAAGATTTCCTCTGGGCATATCCC‐3′ (anti‐sense); for sh‐SAPCD2, 5′‐CACCAGGGCACTTTGGTACACTGTCTTCAAGAGAGACAGTGTACCAAAGTGCCCTTTTTTTG‐3′ (sense) and 5′‐GATCCAAAAAAAGGGCACTTTGGTACACTGTCTCTCTTGAAGACAGTGTACCAAAGTGCCCT‐3′ (anti‐sense)^34^; for sh‐NC, 5′‐CACCGTTCTCCGAACGTGTCACGTTTCAAGAGAACGTGACACGTTCGGAGAATTTTTTG‐3′ (sense) and 5′‐GATCCAAAAAATTCTCCGAACGTGTCACGTTCTCTTGAAACGTGACACGTTCGGAGAAC‐3′ (anti‐sense).

pcDNA3.1‐PXN‐AS1‐L, pcDNA3.1‐SAPCD2, or pcDNA3.1 was transfected into indicted NPC cells by Lipofectamine 3000 (Invitrogen) following the instruction. Seventy‐two hours later, the transfected cells were treated with neomycin for 4 weeks. PXN‐AS1‐L or SAPCD2 stably overexpressed NPC cells were selected and confirmed by qPCR or western blot. sh‐PXN‐AS1‐L, sh‐SAPCD2, or sh‐NC was transfected into indicted NPC cells by Lipofectamine 3000. Seventy‐two hours later, the transfected cells were treated with hygromycin for 4 weeks. PXN‐AS1‐L or SAPCD2 stably silenced NPC cells were selected and confirmed by qPCR or western blot. sh‐SAPCD2 or sh‐NC was transfected into PXN‐AS1‐L stably overexpressed SUNE1 cells using Lipofectamine 3000. Seventy‐two hours later, the transfected cells were treated with neomycin and hygromycin for 4 weeks. PXN‐AS1‐L stably overexpressed and concurrently SAPCD2 stably silenced SUNE1 cells were selected and confirmed by qPCR and western blot.

### 2.4 RNA isolation, reverse transcription, and real‐time quantitative polymerase chain reaction (qPCR)

RNA was isolated from indicated tissues and cells with RNAiso Plus (Takara, Dalian, China) following the protocol. After being treated with DNase I (Takara) to remove DNA, the purified RNA was used to perform reverse transcription with the PrimeScript^™^ II 1st Strand cDNA Synthesis Kit (Takara) following the instruction. Next, the cDNA was used to perform real‐time quantitative polymerase chain reaction (qPCR) with SYBR^®^ Premix Ex Taq^™^ II (Takara) on 7900HT Fast Real‐Time PCR System (Applied Biosystems, Foster City, CA) following the instructions. The sequences of the qPCR primers were: for PXN‐AS1‐L, 5′‐ACCCATCCTCAACTACCCC‐3′ (sense) and 5′‐ACTTCGTCTGTGCCTTCTGC‐3′ (anti‐sense)^33^; for SAPCD2, 5′‐CAGGAGGTGACCGAGAAGA‐3′ (sense) and 5′‐TGAAGGTGGAATCCAGAGG‐3′ (anti‐sense); for PXN, 5′‐TATCTCAGCCCTCAACACGC‐3′ (sense) and 5′‐GGCAGAAGGCACAGACGAA‐3′ (anti‐sense)^33^; for GAPDH, 5′‐GGTCTCCTCTGACTTCAACA‐3′ (sense) and 5′‐GTGAGGGTCTCTCTCTTCCT‐3′ (anti‐sense). GAPDH was used as endogenous control and the comparative Ct method was employed to quantify the expression of RNA.

### 2.5 Cell proliferation assay

Cell Counting Kit‐8 (CCK‐8) and Ethynyl deoxyuridine (EdU) incorporation experiments were employed to determine cell proliferation ability. For CCK‐8 assay, 3000 cells were seeded per well into 96‐well plates. After culturing for indicted time, cell proliferation was evaluated using the Cell Counting Kit‐8 (Dojindo Laboratories) in accordance with the instruction. The absorbance values at 450 nm at each time point were collected to plot cell proliferation curves. EdU incorporation experiment was performed with the Cell‐Light^™^ EdU Apollo^®^643 In Vitro Imaging Kit (RiboBio, Guangzhou, China) in accordance with the instruction. The results were counted using Zeiss AxioPhot Photomicroscope (Carl Zeiss, Oberkochen, Germany) via collecting at least 5 random fields.

### 2.6 Cell migration and invasion assays

Transwell migration and invasion assays were employed to determine cell migration and invasion ability. Briefly, 50 000 indicated NPC cells re‐suspended in 200 μL serum‐free medium were seeded into the upper chamber of a transwell insert without (migration) or with (invasion) pre‐coated matrigel. Complete medium was added into the bottom wells. After culturing for 48 hours, the cells remain in the upper chamber were removed. The cells migrated or invaded through the chambers were fixed using methyl alcohol, stained using crystal violet, and counted using Zeiss AxioPhot Photomicroscope via collecting at least 5 random fields.

### 2.7 RNA pull‐down

PXN‐AS1‐L was in vitro transcribed and biotin‐labeled from pSPT19‐PXN‐AS1‐L using the Biotin RNA Labeling Mix (Roche) and T7 RNA polymerase (Roche). After being treated with DNase I (Takara) to remove DNA and purified using RNeasy Mini Kit (Qiagen, Shenzhen, China), 3 µg of purified RNA was incubated with 1 mg of whole‐cell lysate from SUNE1 cells for 1 hour at 25°C. Next, the complexes were extracted by streptavidin agarose beads (Invitrogen) and the RNA present in the pull‐down material was detected by qPCR as described above.

### 2.8 RNA immunoprecipitation

SUNE1 cells were used to carry out RNA immunoprecipitation (RIP) assay with the Magna RIP RNA‐Binding Protein Immunoprecipitation Kit (Millipore, Bedford, MA) and an AGO2 specific antibody (Millipore) following the instructions. RIP‐derived RNA was detected by qPCR as described above.

### 2.9 Dual luciferase reporter assay

pmirGLO or pmirGLO‐SAPCD2 was co‐transfected with pcDNA3.1‐PXN‐AS1‐L or pcDNA3.1 into SUNE1 cells by Lipofectamine 3000 (Invitrogen). After culturing for 48 hours, the Firefly luciferase activity and Renilla luciferase activity were detected by the Dual‐Luciferase Reporter Assay System (Promega) in accordance with the instruction.

### 2.10 Western blot

Protein expression was quantified by western blot. Total proteins were isolated from indicated NPC cells using RIPA lysis buffer (Beyotime, Shanghai, China). Equal amount of proteins was separated using 12% sodium dodecyl sulfate‐polyacrylamide gel electrophoresis, followed by being transferred onto nitrocellulose membrane (Millipore). After being blocked using 5% nonfat milk, the membranes were incubated with SAPCD2 (Abcam, Hong Kong, China) or GAPDH (Cell Signaling Technology, Boston, MA) specifically primary antibodies. After 3 washes, the membranes were further incubated with IRdye 700‐conjugated goat anti‐mouse IgG or IRdye 800‐conjugated goat anti‐rabbit IgG (Li‐Cor, Lincoln, NE). After 3 washes, the membranes were detected on an Odyssey infrared scanner (Li‐Cor).

### 2.11 Xenograft assays

A total of 1 × 10^7^ indicted NPC cells re‐suspended in 100 μL phosphate buffered saline were subcutaneously injected into the flanks of 4‐ to 5‐week‐old female athymic BALB/C nude mice. Subcutaneous tumor volumes were measured every 3 days by a caliper and calculated following the equation “volume = *a* × *b* ^2^ × 0.5 (*a*, longest diameter; *b*, shortest diameter).” At the 18th day after injection, the mice were sacrificed and subcutaneous tumors were resected and weighed. The Medical Ethics Committee of the People's Hospital of Henan Province (Zhengzhou, China) reviewed and approved the use of mice. Proliferation marker proliferating cell nuclear antigen (PCNA) immunohistochemistry (IHC) staining was performed on paraffin sections of these subcutaneous tumors with a PCNA primary antibody (Cell Signaling Technology) and a horseradish peroxidase‐conjugated IgG (Beyotime). The proteins in situ were visualized using 3,3‐diaminobenzidine. Cell apoptosis of subcutaneous tumors was detected by terminal deoxynucleotidyl transferase‐mediated dUTP nick end labelling (TUNEL) assay using the In Situ Cell Death Detection Kit (Roche) in accordance with the instruction. The results were collected using Zeiss AxioPhot Photomicroscope and quantified via counting at least 5 random fields.

### 2.12 Statistical analysis

The GraphPad Prism Software was employed to carry out statistical analyses. For comparisons, Mann‐Whitney test, Log‐rank test, Pearson chi‐square test, one‐way ANOVA followed by Dunnett's multiple comparison tests, Student's *t* test, Spearman correlation analysis, or Kruskal‐Wallis test was performed as indicated. Significant difference was defined at *P* < 0.05.

## 3 RESULTS

### 3.1 PXN‐AS1‐L is upregulated in NPC and correlated with poor survival of NPC patients

**Figure 1:**
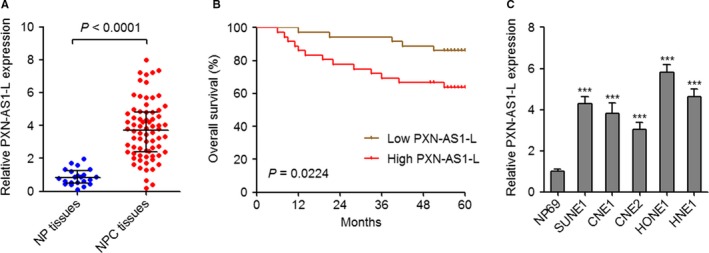
PXN‐AS1‐L is upregulated in nasopharyngeal carcinoma (NPC) and associated with poor survival. A, PXN‐AS1‐L expression levels in 72 NPC tissues and 22 noncancerous nasopharyngeal (NP) tissues were determined by qPCR *P* < 0.0001 by Mann‐Whitney test. B, Kaplan‐Meier analysis of the correlation between PXN‐AS1‐L expression level and overall survival of these 72 NPC patients. The median PXN‐AS1‐L expression level was used as the cutoff. *P* = 0.0224 by Log‐rank test. C, PXN‐AS1‐L expression levels in normal NP epithelium cell line NP69 and NPC cell lines SUNE1, CNE1, CNE2, HONE1, and HNE1 were determined by qPCR. Results are displayed as mean ± SD from 3 independent experiments. ***P < 0.001 by one‐way ANOVA followed by Dunnett's multiple comparison tests

To investigate the expression pattern of PXN‐AS1‐L in NPC, we measured PXN‐AS1‐L expression in 72 NPC tissues and 22 noncancerous NP tissues by qPCR. As displayed in Figure 1A, PXN‐AS1‐L is markedly upregulated in NPC tissues compared to NP tissues. Analyzing the correlation between PXN‐AS1‐L expression levels and clinicopathologic characteristics showed that high expression levels of PXN‐AS1‐L is positively associated with advanced clinical stages and lymph node metastasis (N classification) in these 72 NPC cases (Table 1). Moreover, survival analysis revealed that NPC patients with higher PXN‐AS1‐L expression levels have shorter survival time than those of NPC patients with lower PXN‐AS1‐L expression levels (Figure 1B). PXN‐AS1‐L expression levels in normal NP epithelium cell line NP69 and NPC cell lines SUNE1, CNE1, CNE2, HONE1, and HNE1 were measured by qPCR. The results demonstrated that PXN‐AS1‐L expression levels are elevated in NPC cell lines compared to normal NP epithelium cell line (Figure 1C). Collectively, these data suggested that PXN‐AS1‐L is upregulated in NPC and correlated with advanced clinical stage and poor prognosis of NPC patients, which implied that PXN‐AS1‐L may be involved in the development of NPC.

**Table 1:** Correlation between expression of PXN‐AS1‐L and the clinicopathologic characteristics in nasopharyngeal carcinoma

Characteristics	n	PXN‐AS1‐L expression		*P*‐value
		High	Low	
Age (y)				0.637
>45	34	16	18	
≤45	38	20	18	
Gender				0.448
Male	49	23	26	
Female	23	13	10	
Clinical stage				0.018
I‐II	34	12	22	
III‐IV	38	24	14	
T classification				0.326
T1‐T2	46	21	25	
T3‐T4	26	15	11	
N classification				0.026
N0‐N1	47	19	28	
N2‐N3	25	17	8	
Distant metastasis				0.354
M0	67	32	35	
M1	5	4	1	

Note

*P*‐value was determined by Pearson chi‐square tests.

### 3.2 Overexpression of PXN‐AS1‐L promotes NPC cell proliferation, migration, and invasion

**Figure 2:**
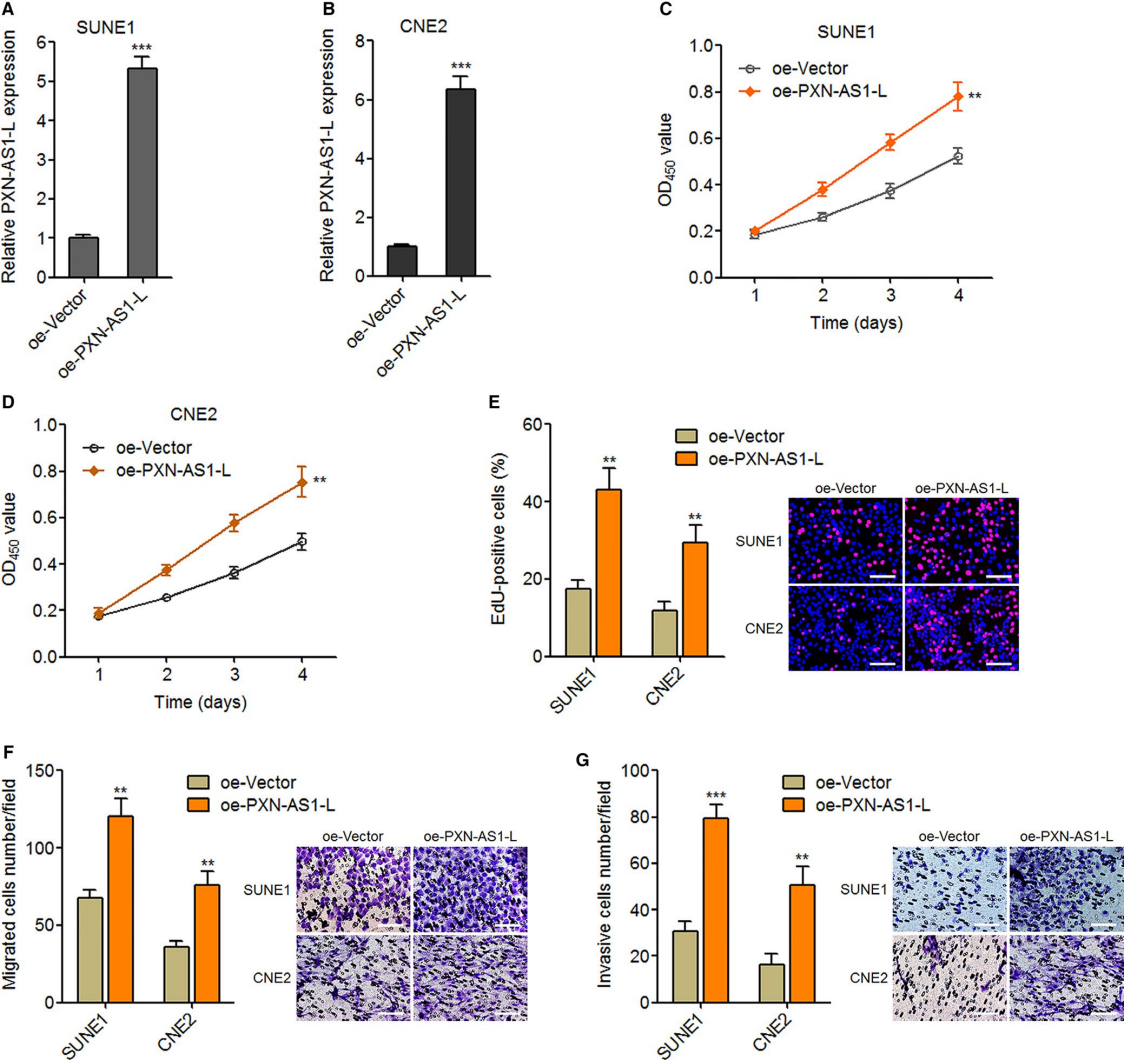
Overexpression of PXN‐AS1‐L promotes nasopharyngeal carcinoma cell proliferation, migration, and invasion. A, PXN‐AS1‐L expression levels in PXN‐AS1‐L stably overexpressed and control SUNE1 cells were determined by qPCR. B, PXN‐AS1‐L expression levels in PXN‐AS1‐L stably overexpressed and control CNE2 cells were determined by qPCR. C, Cell proliferation of PXN‐AS1‐L stably overexpressed and control SUNE1 cells was determined by Cell Counting Kit‐8 (CCK‐8) assay. D, Cell proliferation of PXN‐AS1‐L stably overexpressed and control CNE2 cells was determined by CCK‐8 assay. E, Cell proliferation of PXN‐AS1‐L stably overexpressed and control SUNE1 and CNE2 cells was determined by ethynyl deoxyuridine (EdU) incorporation assay. Scale bars, 100 μm. F, Cell migration of PXN‐AS1‐L stably overexpressed and control SUNE1 and CNE2 cells was determined by transwell migration assay. Scale bars, 100 μm. G, Cell invasion of PXN‐AS1‐L stably overexpressed and control SUNE1 and CNE2 cells was determined by transwell invasion assay. Scale bars, 100 μm. Results are displayed as mean ± SD from 3 independent experiments. **P < 0.01, ***P < 0.001 by Student's *t* test

To explore the biological roles of PXN‐AS1‐L in NPC, we constructed PXN‐AS1‐L stably overexpressed SUNE1 and CNE2 cells through transfecting PXN‐AS1‐L overexpression plasmid. The overexpression efficiencies were confirmed by qPCR (Figure 2A,B). CCK‐8 assays demonstrated that overexpression of PXN‐AS1‐L promotes SUNE1 and CNE2 cell proliferation (Figure 2C,D). The pro‐proliferative roles of PXN‐AS1‐L in SUNE1 and CNE2 cells were further confirmed by EdU incorporation experiments (Figure 2E). Transwell migration experiments demonstrated that overexpression of PXN‐AS1‐L promotes SUNE1 and CNE2 cell migration (Figure 2F). Transwell invasion experiments displayed that overexpression of PXN‐AS1‐L promotes SUNE1 and CNE2 cell invasion (Figure 2G). Collectively, these data demonstrated that overexpression of PXN‐AS1‐L promotes NPC cell proliferation, migration, and invasion.

### 3.3 Silencing of PXN‐AS1‐L suppresses NPC cell proliferation, migration, and invasion

**Figure 3:**
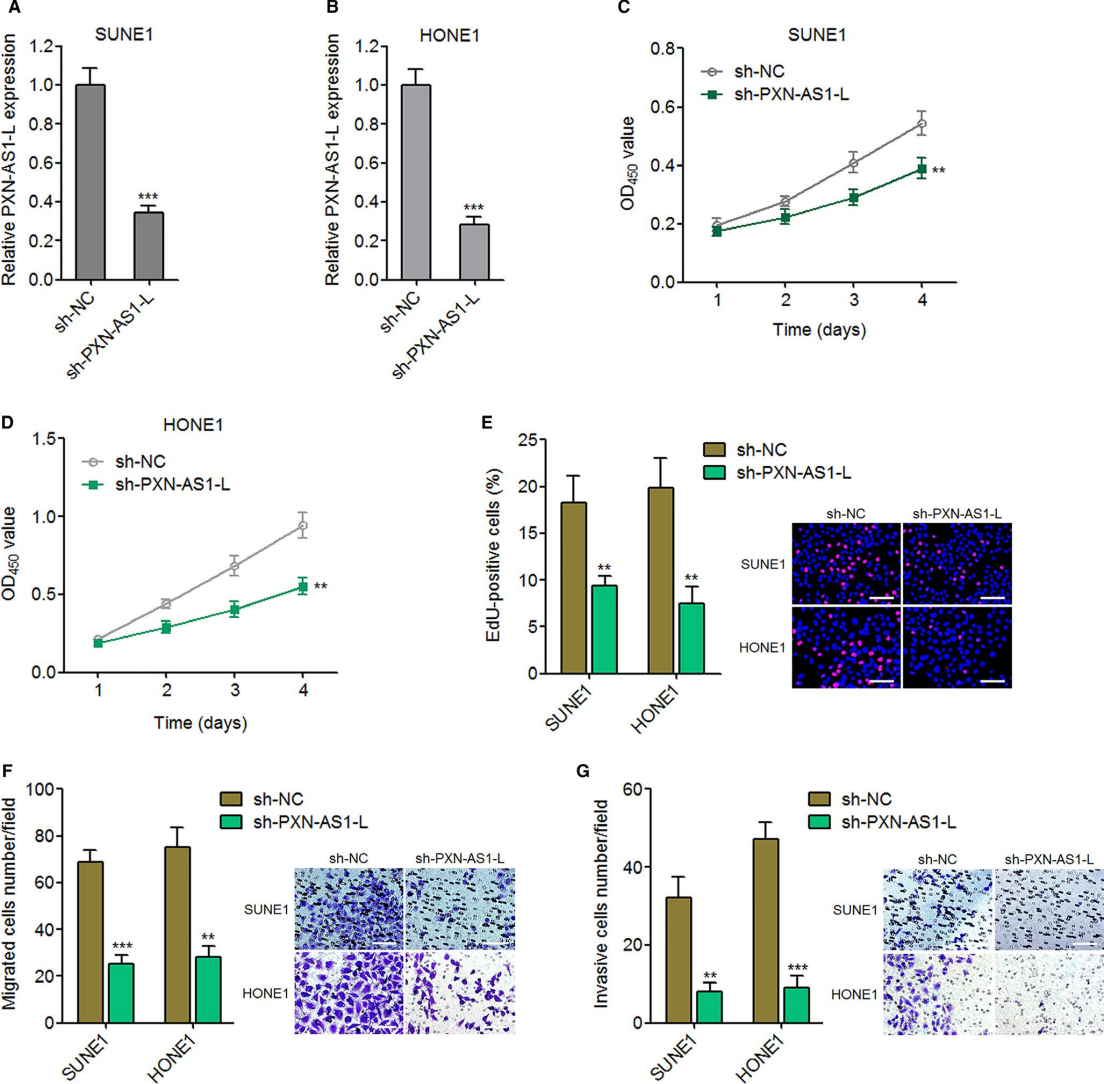
Silencing of PXN‐AS1‐L suppresses nasopharyngeal carcinoma cell proliferation, migration, and invasion. A, PXN‐AS1‐L expression levels in PXN‐AS1‐L stably silenced and control SUNE1 cells were determined by qPCR. B, PXN‐AS1‐L expression levels in PXN‐AS1‐L stably silenced and control HONE1 cells were determined by qPCR. C, Cell proliferation of PXN‐AS1‐L stably silenced and control SUNE1 cells was determined by Cell Counting Kit‐8 (CCK‐8) assay. D, Cell proliferation of PXN‐AS1‐L stably silenced and control HONE1 cells was determined by CCK‐8 assay. E, Cell proliferation of PXN‐AS1‐L stably silenced and control SUNE1 and HONE1 cells was determined by ethynyl deoxyuridine (EdU) incorporation assay. Scale bars, 100 μm. F, Cell migration of PXN‐AS1‐L stably silenced and control SUNE1 and HONE1 cells was determined by transwell migration assay. Scale bars, 100 μm. G, Cell invasion of PXN‐AS1‐L stably silenced and control SUNE1 and HONE1 cells was determined by transwell invasion assay. Scale bars, 100 μm. Results are displayed as mean ± SD from 3 independent experiments. **P < 0.01, ***P < 0.001 by Student's *t* test

For completely determining the oncogenic roles of PXN‐AS1‐L in NPC, we further constructed PXN‐AS1‐L stably silenced SUNE1 and HONE1 cells through transfecting PXN‐AS1‐L specific shRNA. The silencing efficiencies were confirmed by qPCR (Figure 3A,B). CCK‐8 experiments revealed that silencing of PXN‐AS1‐L suppresses SUNE1 and HONE1 cell proliferation (Figure 3C,D). The proliferation suppressive roles of PXN‐AS1‐L silencing in SUNE1 and HONE1 cells were further confirmed using EdU incorporation assays (Figure 3E). Transwell migration experiments demonstrated that silencing of PXN‐AS1‐L represses SUNE1 and HONE1 cell migration (Figure 3F). Transwell invasion assays demonstrated that silencing of PXN‐AS1‐L suppresses SUNE1 and HONE1 cell invasion (Figure 3G). Collectively, these results showed that silencing of PXN‐AS1‐L represses NPC cell proliferation, migration, and invasion.

### 3.4 PXN‐AS1‐L upregulates SAPCD2

**Figure 4:**
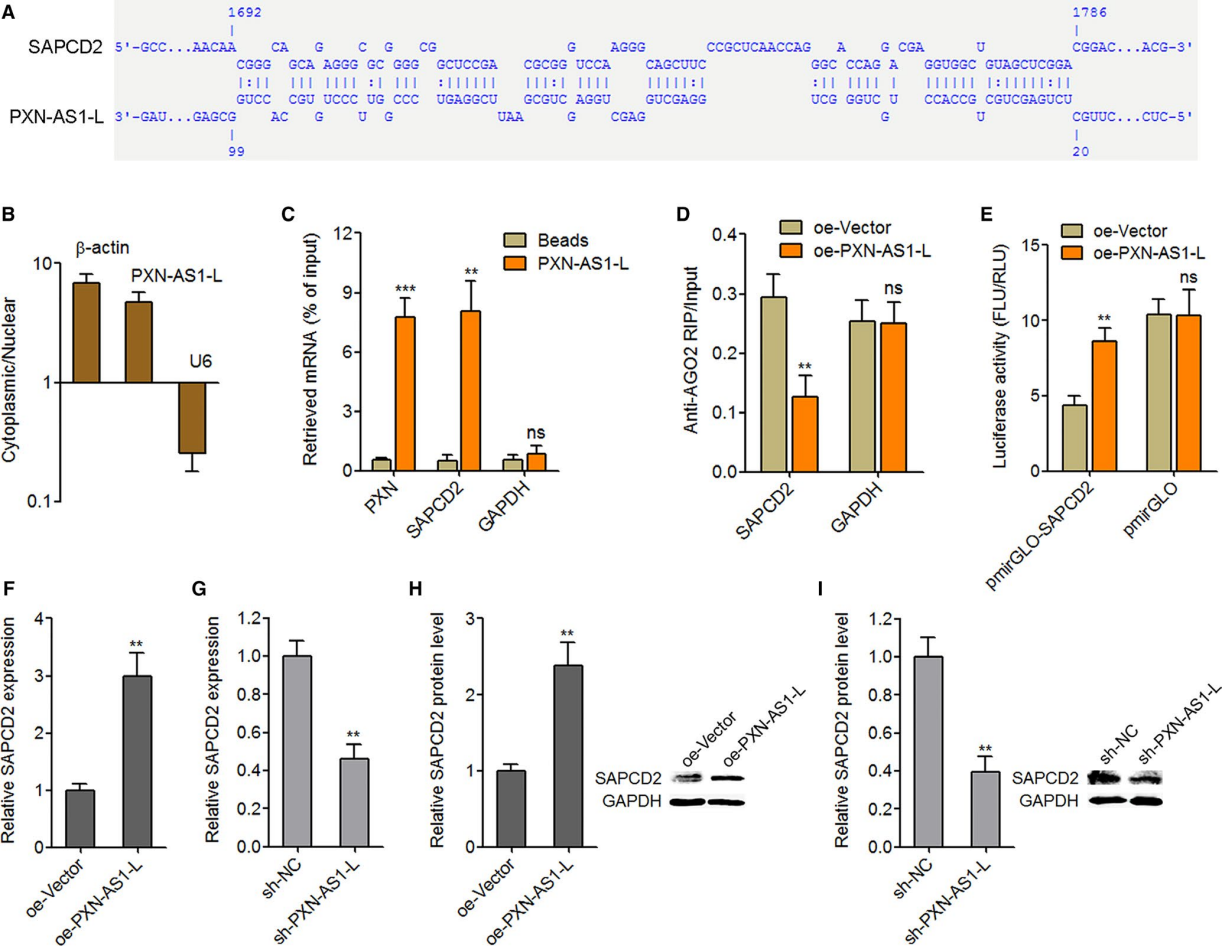
PXN‐AS1‐L upregulates SAPCD2. A, Schematic diagram of the predicted interaction sequences between PXN‐AS1‐L and SAPCD2 mRNA by IntaRNA. B, Subcellular localization of PXN‐AS1‐L was determined by nuclear and cytoplasmic RNA isolation followed by qPCR. C, In vitro transcribed biotin‐labeled PXN‐AS1‐L was incubated with SUNE1 cell extracts, enriched by streptavidin beads, and washed. The enrichment of SAPCD2, PXN and GAPDH mRNA was determined by qPCR. PXN was used as a positive control. D, After transient transfection of PXN‐AS1‐L overexpression or control plasmids into SUNE1 cells, RNA immunoprecipitation experiments were performed using AGO specific antibody. The enrichment of SAPCD2 and GAPDH mRNA was determined by qPCR. E, PXN‐AS1‐L overexpression or control plasmids were co‐transfected with luciferase reporters containing SAPCD2 3′‐untranslated region (3′UTR) (pmirGLO‐SAPCD2) or control (pmirGLO) into SUNE1 cells. Dual luciferase reporter assays were performed to investigate the effects of PXN‐AS1‐L on SAPCD2 3′UTR activity. Results are shown as the ratio of Firefly luciferase activity (FLU) to Renilla luciferase activity (RLU). F, SAPCD2 mRNA levels in PXN‐AS1‐L stably overexpressed and control SUNE1 cells were determined by qPCR. G, SAPCD2 mRNA levels in PXN‐AS1‐L stably silenced and control HONE1 cells were determined by qPCR. H, SAPCD2 protein levels in PXN‐AS1‐L stably overexpressed and control SUNE1 cells were determined by western blot. I, SAPCD2 protein levels in PXN‐AS1‐L stably silenced and control HONE1 cells were determined by western blot. Results are displayed as mean ± SD from 3 independent experiments. **P < 0.01, ***P < 0.001, ns, not significant, by Student's *t*‐test

To explore the molecular mechanisms responsible for the oncogenic roles of PXN‐AS1‐L in NPC, we searched The Cancer Genome Atlas (TCGA) dataset to identify the genes whose expression was correlated with PXN‐AS1‐L using TANRIC (http://ibl.mdanderson.org/tanric/_design/basic/index.html). *SAPCD2* (C9orf140) is one of the most positively correlated genes (Table S1). SAPCD2, also known as p42.3 or C9orf140, is previously shown to exert oncogenic roles in melanoma, gastric cancer, HCC, and colorectal cancer.^34^, ^35^, ^36^, ^37^ Intriguingly, we further predicted a long interaction region between PXN‐AS1‐L and 3′UTR of SAPCD2 mRNA by IntaRNA (http://rna.informatik.uni-freiburg.de/IntaRNA/Input.jsp) (Figure 4A). Furthermore, PXN‐AS1‐L is found to be mainly localized in cytoplasm of NPC cells (Figure 4B), which supported the potential regulatory roles between PXN‐AS1‐L and SAPCD2 mRNA in cytoplasm. Therefore, we further investigated whether SAPCD2 is a downstream target of PXN‐AS1‐L in NPC. RNA pulldown experiments demonstrated that SAPCD2 mRNA is specifically enriched by in vitro‐transcribed biotin‐labeled PXN‐AS1‐L (Figure 4C), which supported the physical binding between PXN‐AS1‐L and SAPCD2 mRNA. microRNAs (miRNAs) are known to form RNA‐induced silencing complex with AGO2 to bind the 3′UTR of target mRNAs and induce translational repression and/or target mRNAs degradation.^38^, ^39^, ^40^, ^41^ Therefore, we further investigated whether PXN‐AS1‐L regulates the effects of miRNAs‐AGO2 complex on SAPCD2 3′UTR. RIP experiments displayed that overexpression of PXN‐AS1‐L reduced the binding of AGO2 to 3′UTR of SAPCD2 mRNA (Figure 4D). SAPCD2 3′UTR containing the predicted PXN‐AS1‐L interaction region was cloned into luciferase reporter downstream of firefly luciferase. Dual luciferase reporter assays displayed that overexpression of PXN‐AS1 upregulated the luciferase activity of SAPCD2 3′UTR (Figure 4E). These data suggested that interaction between PXN‐AS1‐L and SAPCD2 3′UTR protects SAPCD2 3′UTR from miRNAs‐AGO2 complex induced translational repression and/or degradation. The mRNA expression levels of SAPCD2 in PXN‐AS1‐L stably overexpressed SUNE1 cells and PXN‐AS1‐L stably silenced HONE1 cells were measured using qPCR. The results displayed that overexpression of PXN‐AS1 elevated SAPCD2 mRNA levels (Figure 4F), and conversely silencing of PXN‐AS1‐L reduced SAPCD2 mRNA levels (Figure 4G). Furthermore, the protein levels of SAPCD2 in PXN‐AS1‐L stably overexpressed SUNE1 cells and PXN‐AS1‐L stably silenced HONE1 cells were measured using western blot. As displayed in Figure 4H,I, overexpression of PXN‐AS1 elevated SAPCD2 protein levels and conversely silencing of PXN‐AS1‐L reduced SAPCD2 protein levels. Taken together, these results showed that PXN‐AS1‐L interacts with SAPCD2 3′UTR, protects SAPCD2 3′UTR from miRNAs‐AGO2 complex induced translational repression and degradation, and upregulates the expression of SAPCD2.

### 3.5 The expression of SAPCD2 is positively associated with PXN‐AS1‐L in NPC tissues

**Figure 5:**
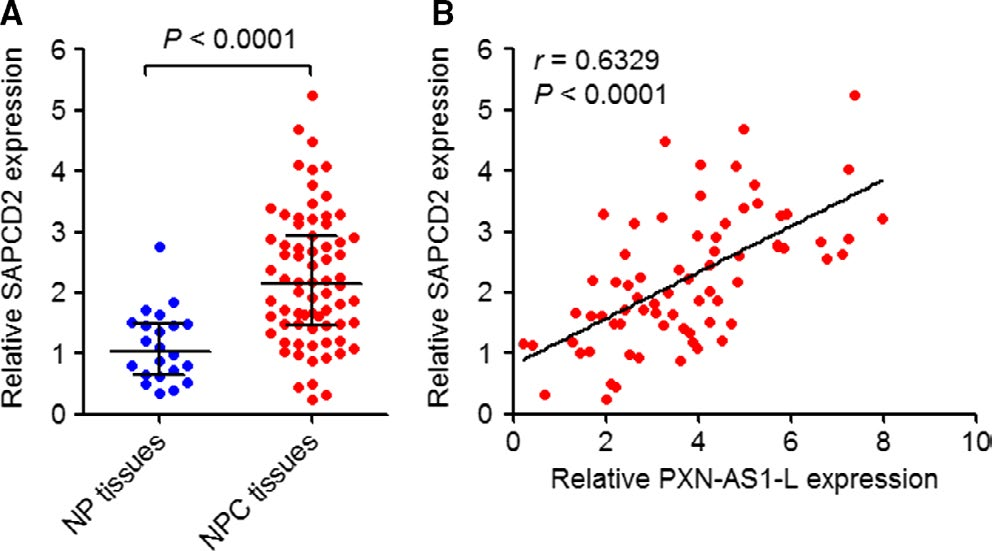
The expression correlation between PXN‐AS1‐L and SAPCD2 in nasopharyngeal carcinoma (NPC) tissues. A, SAPCD2 expression levels in 72 NPC tissues and 22 noncancerous nasopharyngeal (NP) tissues were determined by qPCR *P* < 0.0001 by Mann‐Whitney test. B, The correlation between PXN‐AS1‐L and SAPCD2 expression levels in these 72 NPC tissues. *r* = 0.6329, *P* < 0.0001 by Spearman's correlation analysis

SAPCD2 expression levels in the same 72 NPC tissues and 22 noncancerous NP tissues used in Figure 1A were measured by qPCR. As displayed in Figure 5A, PXN‐AS1‐L is consistently increased in NPC tissues compared to NP tissues. Moreover, the expression of SAPCD2 is significantly positively correlated with that of PXN‐AS1‐L in these 72 NPC tissues (*r* = 0.6329, *P* < 0.0001) (Figure 5B).

### 3.6 SAPCD2 promotes NPC cell proliferation, migration, and invasion

**Figure 6:**
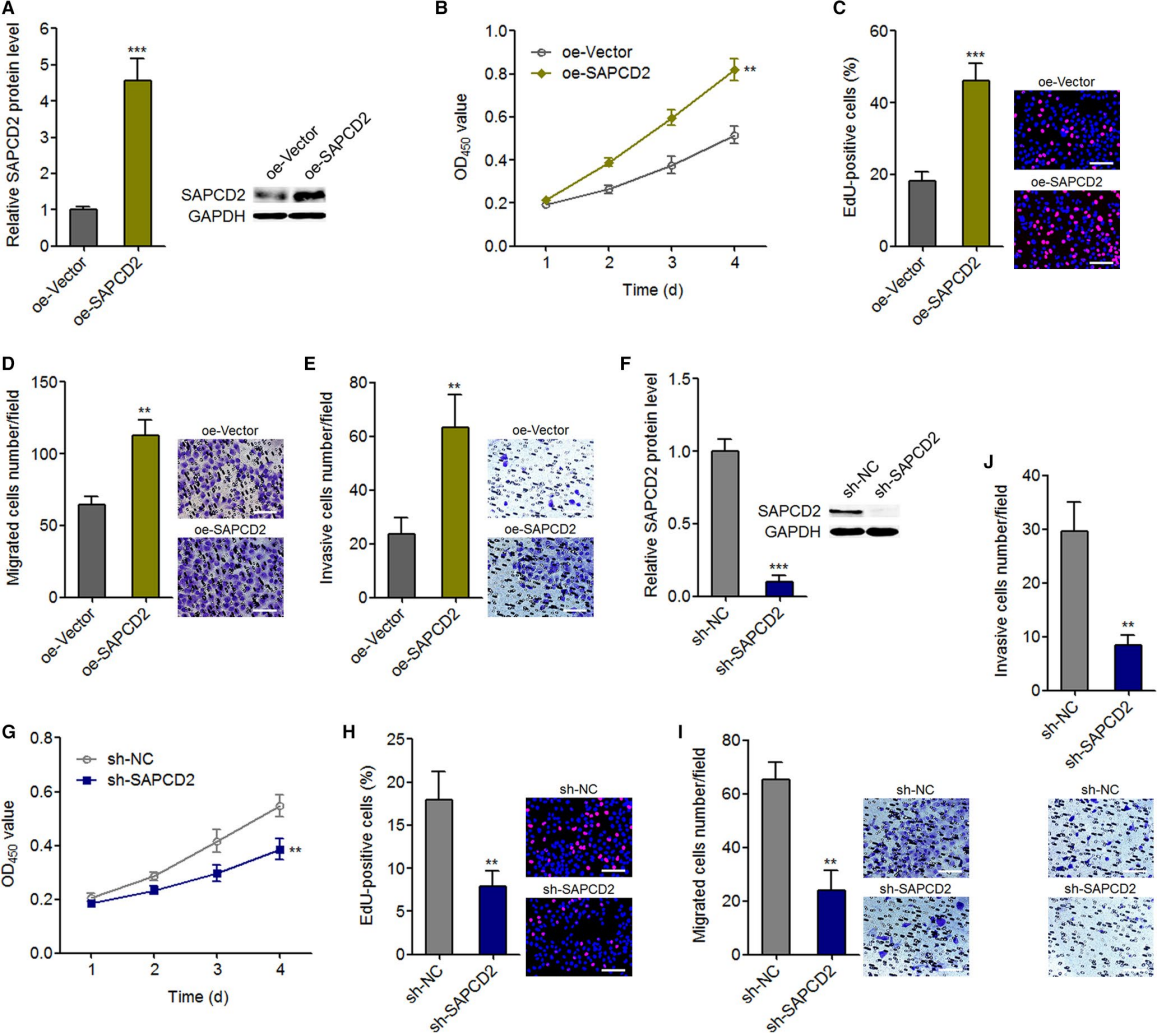
SAPCD2 promotes nasopharyngeal carcinoma cell proliferation, migration, and invasion. A, SAPCD2 protein levels in SAPCD2 stably overexpressed and control SUNE1 cells were determined by western blot. B, Cell proliferation of SAPCD2 stably overexpressed and control SUNE1 cells was determined by Cell Counting Kit‐8 (CCK‐8) assay. C, Cell proliferation of SAPCD2 stably overexpressed and control SUNE1 cells was determined by ethynyl deoxyuridine (EdU) incorporation assay. Scale bars, 100 μm. D, Cell migration of SAPCD2 stably overexpressed and control SUNE1 cells was determined by transwell migration assay. Scale bars, 100 μm. E, Cell invasion of SAPCD2 stably overexpressed and control SUNE1 cells was determined by transwell invasion assay. Scale bars, 100 μm. F, SAPCD2 protein levels in SAPCD2 stably silenced and control SUNE1 cells were determined by western blot. G, Cell proliferation of SAPCD2 stably silenced and control SUNE1 cells was determined by CCK‐8 assay. H, Cell proliferation of SAPCD2 stably silenced and control SUNE1 cells was determined by EdU incorporation assay. Scale bars, 100 μm. I, Cell migration of SAPCD2 stably silenced and control SUNE1 cells was determined by transwell migration assay. Scale bars, 100 μm. J, Cell invasion of SAPCD2 stably silenced and control SUNE1 cells was determined by transwell invasion assay. Scale bars, 100 μm. Results are displayed as mean ± SD from 3 independent experiments. **P < 0.01, ***P < 0.001 by Student's *t*‐test

Although SAPCD2 is revealed to function as an oncogene in melanoma, gastric cancer, HCC, and colorectal cancer, the biological roles of SAPCD2 in NPC are still unknown. To determine the biological roles of SAPCD2 in NPC, we constructed SAPCD2 stably overexpressed SUNE1 cells through transfecting SAPCD2 overexpression plasmid. The overexpression efficiency was confirmed using western blot (Figure 6A). CCK‐8 and EdU incorporation experiments both demonstrated that overexpression of SAPCD2 promotes cell proliferation (Figure 6B,C). Transwell migration experiments demonstrated that overexpression of SAPCD2 promotes cell migration (Figure 6D). Transwell invasion experiments displayed that overexpression of SAPCD2 promotes cell invasion (Figure 6E). Furthermore, we constructed SAPCD2 stably silenced SUNE1 cells through transfecting SAPCD2 specific shRNA. The silencing efficiency was confirmed using western blot (Figure 6F). CCK‐8 and EdU incorporation experiments demonstrated that silencing of SAPCD2 suppresses cell proliferation (Figure 6G,H). Transwell migration experiments displayed that silencing of SAPCD2 suppresses cell migration (Figure 6I). Transwell invasion assays demonstrated that silencing of SAPCD2 suppresses cell invasion (Figure 6J). Taken together, these results showed that consistent with PXN‐AS1‐L, SAPCD2 also promotes NPC cell proliferation, migration, and invasion.

### 3.7 The oncogenic roles of PXN‐AS1‐L in NPC are dependent on the regulation of SAPCD2

**Figure 7:**
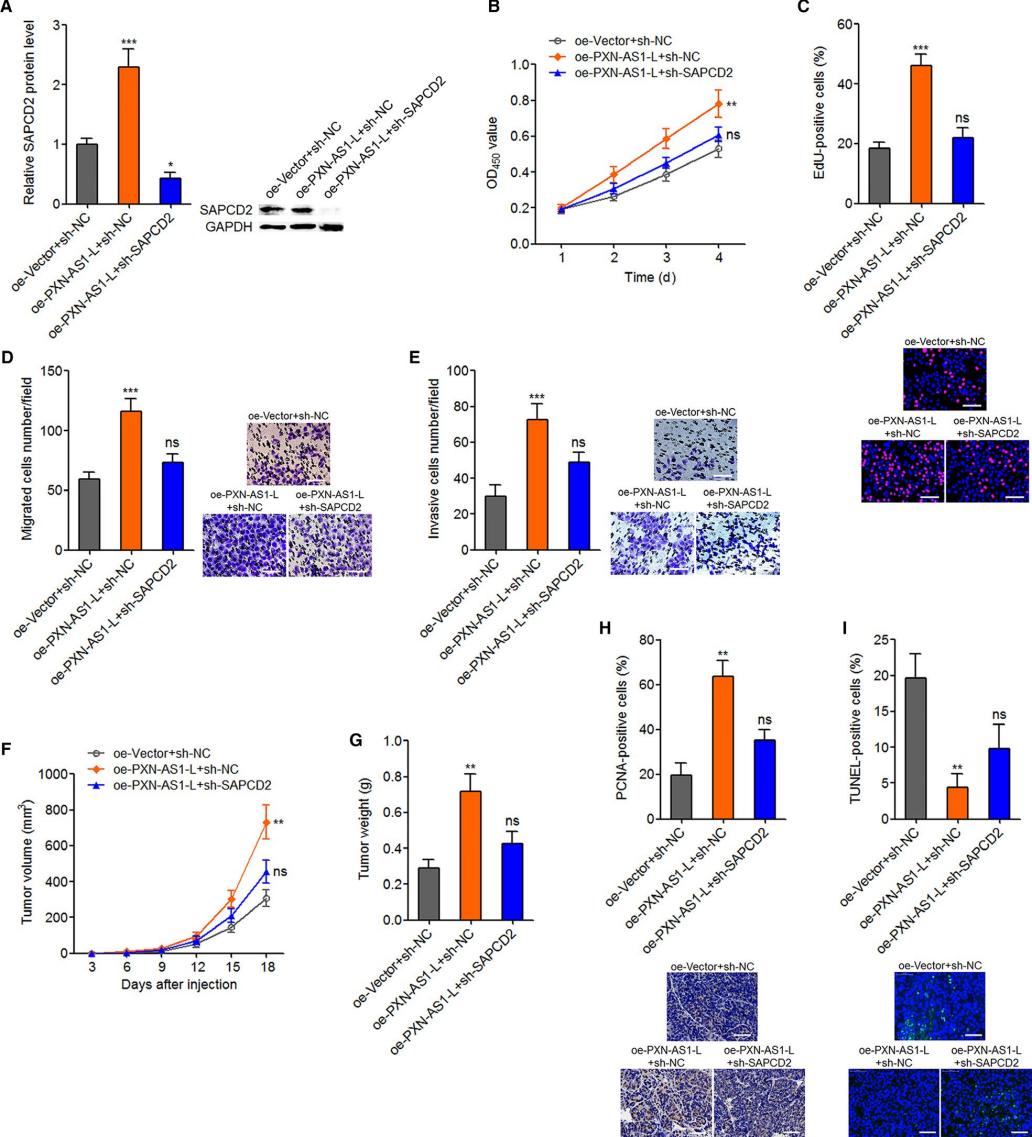
SAPCD2 knockdown attenuates the oncogenic roles of PXN‐AS1‐L overexpression in nasopharyngeal carcinoma. A, SAPCD2 protein levels in PXN‐AS1‐L stably overexpressed and concurrently SAPCD2 stably silenced SUNE1 cells were determined by western blot. B, Cell proliferation of PXN‐AS1‐L overexpressed and concurrently SAPCD2 silenced SUNE1 cells was determined by Cell Counting Kit‐8 assay. C, Cell proliferation of PXN‐AS1‐L overexpressed and concurrently SAPCD2 silenced SUNE1 cells was determined by ethynyl deoxyuridine (EdU) incorporation assay. Scale bars, 100 μm. D, Cell migration of PXN‐AS1‐L overexpressed and concurrently SAPCD2 silenced SUNE1 cells was determined by transwell migration assay. Scale bars, 100 μm. E, Cell invasion of PXN‐AS1‐L overexpressed and concurrently SAPCD2 silenced SUNE1 cells was determined by transwell invasion assay. For A‐E, results are displayed as mean ± SD from 3 independent experiments. *P < 0.05, **P < 0.01, ***P < 0.001, ns, not significant, by one‐way ANOVA followed by Dunnett's multiple comparison tests. F, PXN‐AS1‐L overexpressed and concurrently SAPCD2 silenced SUNE1 cells were subcutaneously injected into nude mice. Tumor volumes were measured every 3 days. G, The subcutaneous tumors were resected and weighed at the 18th day after injection. H, PCNA immunohistochemistry staining of subcutaneous tumors from G. Scale bars, 50 μm. I, terminal deoxynucleotidyl transferase‐mediated dUTP nick end labelling staining of subcutaneous tumors from G. Scale bars, 50 μm. For F‐I, results are displayed as mean ± SD from 5 mice in each group. **P < 0.01, ns, not significant, by Kruskal‐Wallis test

To determine whether PXN‐AS1‐L exerts its oncogenic roles via regulation of SAPCD2, we stably silenced SAPCD2 expression in PXN‐AS1‐L stably overexpressed SUNE1 cells (Figure 7A). CCK‐8 and EdU incorporation experiments showed that silencing of SAPCD2 attenuated the pro‐proliferative roles of PXN‐AS1‐L overexpression (Figure 7B,C). Transwell migration assays demonstrated that silencing of SAPCD2 attenuated the pro‐migratory roles of PXN‐AS1‐L overexpression (Figure 7D). Transwell invasion experiments showed that silencing of SAPCD2 attenuated the pro‐invasive roles of PXN‐AS1‐L overexpression (Figure 7E). Furthermore, these constructed SUNE1 cells were subcutaneously injected into nude mice. Tumor volumes were measured every 3 days. Subcutaneous tumors were resected and weighed at the 18th day after injection. As displayed in Figure 7F,G, overexpression of PXN‐AS1‐L promotes NPC tumor growth in vivo. Silencing of SAPCD2 attenuates the pro‐growth roles of PXN‐AS1‐L overexpression in vivo. Proliferation marker PCNA IHC staining displayed that overexpression of PXN‐AS1‐L upregulates PCNA expression, which is attenuated by SAPCD2 silencing (Figure 7H). Apoptosis marker TUNEL staining displayed that overexpression of PXN‐AS1‐L reduces the number of apoptotic cells, which is reversed by SAPCD2 silencing (Figure 7I). These data demonstrated that SAPCD2 silencing attenuates both the in vitro and in vivo oncogenic roles of PXN‐AS1‐L in NPC. In addition, we overexpressed SAPCD2 in PXN‐AS1‐L stably silenced SUNE1 cells (Figure S1A). CCK‐8 and EdU incorporation experiments showed that overexpression of SAPCD2 attenuated the proliferation inhibitory roles of PXN‐AS1‐L silencing (Figure S1B,C). Transwell migration assays demonstrated that overexpression of SAPCD2 attenuated the migration inhibitory roles of PXN‐AS1‐L silencing (Figure S1D). Transwell invasion experiments showed that overexpression of SAPCD2 attenuated the invasion inhibitory roles of PXN‐AS1‐L silencing (Figure S1E). These data demonstrated that SAPCD2 overexpression attenuates the tumor suppressive roles of PXN‐AS1‐L silencing in NPC. Collectively, these finding suggested that the oncogenic roles of PXN‐AS1‐L in NPC are dependent on the regulation of SAPCD2.

## 4 DISCUSSION

Yuan et al recently reported that splicing factor MBNL3 modulated the alternative splicing of lncRNA PXN‐AS1, which generated 2 different isoforms of PXN‐AS1.^33^ PXN‐AS1‐L is one of the isoforms which contains the exon 4 and has 863 nucleotides in length, and whereas PXN‐AS1‐S is another isoform which lacks the exon 4 and has 686 nucleotides in length.^33^ They found PXN‐AS1‐L was upregulated in HCC tissues and had oncogenic roles in HCC.^33^ In this study, we focused our attention on lncRNA PXN‐AS1‐L. Using isoform specific primers, we found that PXN‐AS1‐L is also increased in NPC tissues and cell lines compared with noncancerous NP tissues and normal NP epithelium cell line, respectively. Higher expression level of PXN‐AS1‐L is positively correlated with advanced clinical stage, lymph node metastasis, and poor survival of NPC patients. These data implied that PXN‐AS1‐L may be a promising prognostic biomarker for NPC. Multicenter studies enrolling more NPC patients can provide stronger evidences for the application of PXN‐AS1‐L for NPC patients’ prognosis. Furthermore, whether PXN‐AS1‐L is also upregulated in other cancers except HCC and NPC and whether PXN‐AS1‐L is correlated with outcome of other cancers patients need further exploration.

Functional assays revealed that overexpression of PXN‐AS1‐L promotes NPC cell proliferation, migration, and invasion in vitro. PXN‐AS1‐L silencing represses NPC cell proliferation, migration, and invasion in vitro. Furthermore, we also found that overexpression of PXN‐AS1‐L promotes NPC tumor growth in vivo. Therefore, these findings demonstrated that PXN‐AS1‐L acts as an oncogene in NPC. Our findings also implied that PXN‐AS1‐L would be a potential therapeutic target for NPC. Previous report has identified the oncogenic roles of PXN‐AS1‐L in HCC.^33^ Thus, we speculate that PXN‐AS1‐L may be an important oncogene in human cancers. More investigations about the functions of PXN‐AS1‐L in other cancers can validate this speculation.

The molecular mechanisms exerted by lncRNAs are diverse. Using TCGA dataset, we noted that the expression of PXN‐AS1‐L is significantly positively associated with SAPCD2 (*r* = 0.572) in head and neck squamous cell carcinoma. The significant association between PXN‐AS1‐L expression level and SAPCD2 expression level was further verified in NPC tissues (*r* = 0.633). Therefore, we further investigated the regulatory effects between PXN‐AS1‐L and SAPCD2. Our findings revealed that PXN‐AS1‐L upregulated the mRNA and protein levels of SAPCD2 in NPC cells. But SAPCD2 did not regulate the transcript level of PXN‐AS1‐L. Next, we investigated the detailed mechanism mediating the upregulation of SAPCD2 by PXN‐AS1‐L. PXN‐AS1‐L is mainly distributed in cytoplasm. Several cytoplasmic lncRNAs were shown to directly bind mRNAs and regulate the stability and/or translation of target mRNAs.^42^, ^43^ LncRNA BACE1‐AS increased BACE1 mRNA stability and upregulated BACE1 protein expression.^42^ Antisense Uchl1 was reported to promote UCHL1 mRNA translation.^43^ In this study, we also revealed that PXN‐AS1‐L directly bound to SAPCD2 mRNA. Intriguingly, the interaction sites of SAPCD2 mRNA are located at 3′UTR. 3′UTR are well known target sites of miRNAs. Indeed, we found that the interaction between PXN‐AS1‐L and SAPCD2 mRNA decreased the binding of AGO2‐miRNAs silencing complex on SAPCD2 mRNA. Dual luciferase reporter assays also showed that PXN‐AS1‐L increased SAPCD2 mRNA 3′UTR activity. Collectively, our findings suggested that PXN‐AS1‐L interacts with SAPCD2 mRNA 3′UTR and relieves the repressive roles of AGO2‐miRNAs silencing complex on SAPCD2 mRNA stability and translation. The concrete miRNAs involved in the modulation need further investigation. Functional experiments further revealed that silencing of SAPCD2 significantly reversed the oncogenic roles of PXN‐AS1‐L in NPC in vitro and in vivo, which supported that SAPCD2 was an important mediator of the roles of PXN‐AS1‐L in NPC. In this study, we also found that PXN‐AS1‐L interacts with PXN mRNA as reported in HCC.^33^ PXN may be another mediator of the roles of PXN‐AS1‐L in NPC, which needs further investigation. This study identified a novel action mechanism of PXN‐AS1‐L in NPC, which suggested the complex of action mechanisms of lncRNAs in different cancers. More completely investigating the molecular mechanisms of PXN‐AS1‐L will benefit the application of targeting PXN‐AS1‐L in cancer treatment. In this study, we focused on PXN‐AS1‐L. The expression, function, and action mechanism of another isoform PXN‐AS1‐S in NPC need further investigations to completely understand the significances of PXN‐AS1.

In summary, this study found that lncRNA PXN‐AS1‐L is increased in NPC and correlated with poor prognosis of NPC patients. PXN‐AS1‐L promotes NPC cell proliferation, migration, and invasion in vitro, and NPC tumor growth in vivo via upregulating SAPCD2 expression. Targeted inhibition of PXN‐AS1‐L may be a potential anticancer strategy for NPC.

## CONFLICT OF INTEREST

None declared.
