# Supplementary figures and images for: Long noncoding RNA PXN‐AS1‐L promotes the malignancy of nasopharyngeal carcinoma cells via upregulation of SAPCD2
Source: Cancer Med. 2019 Jun 7;8(9):4278–91. doi: 10.1002/cam4.2227 (PMC6675719; doi:10.1002/cam4.2227)

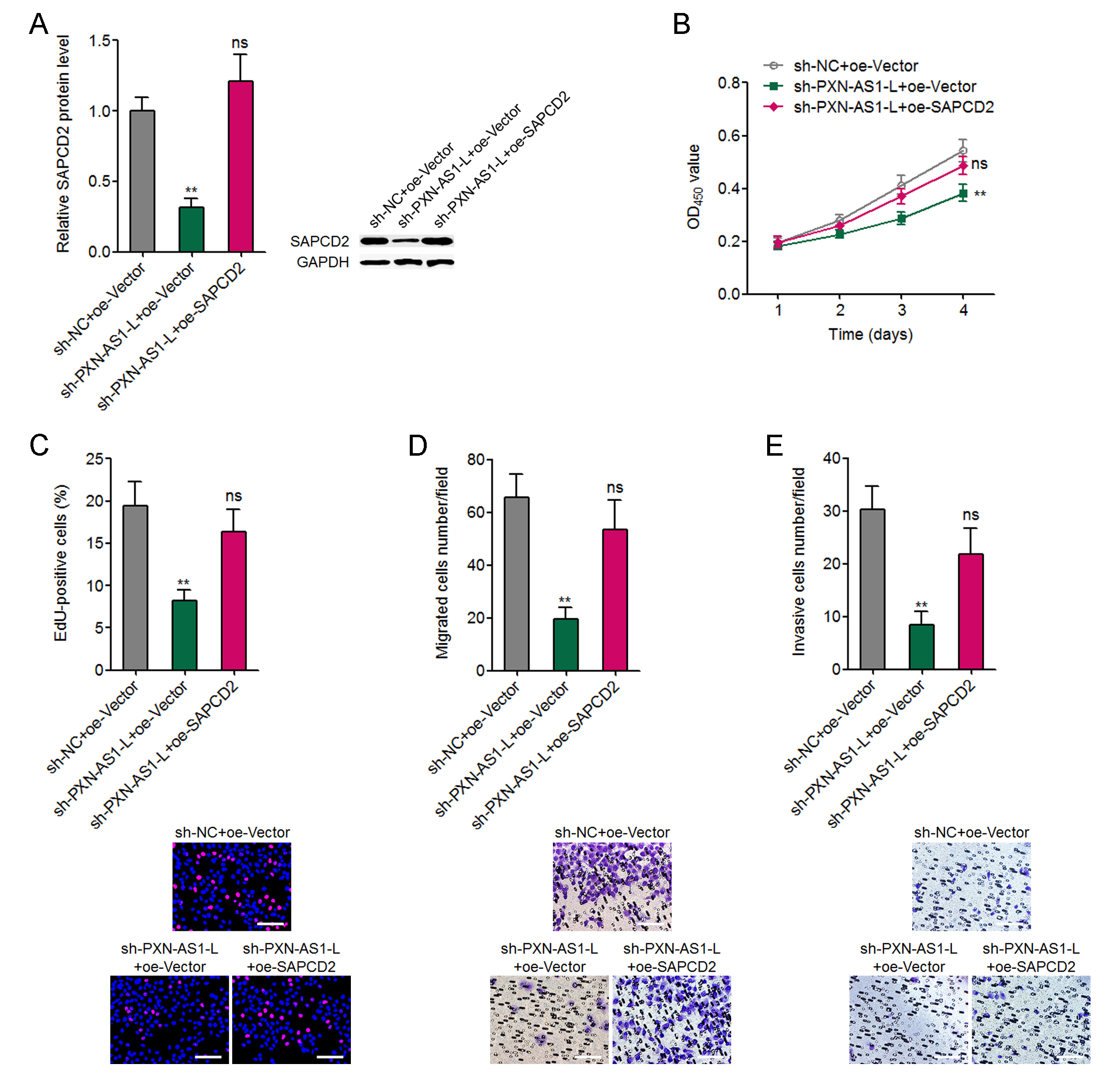

Supplement: Supplementary file 1 [file CAM4-8-4278-s001.tif]
