# Supplementary material for: Long noncoding RNA PXN‐AS1‐L promotes the malignancy of nasopharyngeal carcinoma cells via upregulation of SAPCD2
Source: Cancer Med. 2019 Jun 7;8(9):4278–91. doi: 10.1002/cam4.2227 (PMC6675719; doi:10.1002/cam4.2227)
Supplement: Supplementary file 3 [file CAM4-8-4278-s003.docx]

**Supplementary Figure legends**

**Supplementary Figure 1. SAPCD2 overexpression attenuates the tumor suppressive roles of PXN-AS1-L silencing in NPC.** (A) SAPCD2 overexpression plasmid was transfected into PXN-AS1-L stably silenced SUNE1 cells. SAPCD2 protein levels in the transfected cells were determined by western blot. (B) SAPCD2 overexpression plasmid was transfected into PXN-AS1-L stably silenced SUNE1 cells. Cell proliferation of the transfected cells was determined by CCK-8 assay. (C) SAPCD2 overexpression plasmid was transfected into PXN-AS1-L stably silenced SUNE1 cells. Cell proliferation of the transfected cells was determined by EdU incorporation assay. Scale bars, 100 μm. (D) SAPCD2 overexpression plasmid was transfected into PXN-AS1-L stably silenced SUNE1 cells. Cell migration of the transfected cells was determined by transwell migration assay. Scale bars, 100 μm. (E) SAPCD2 overexpression plasmid was transfected into PXN-AS1-L stably silenced SUNE1 cells. Cell invasion of the transfected cells was determined by transwell invasion assay. Results are displayed as mean ± SD from 3 independent experiments. ***P* < 0.01, ns, not significant, by one-way ANOVA followed by Dunnett's multiple comparison tests.
